# Needed Approaches for Dietary Research in Migraine

**DOI:** 10.1007/s11916-026-01573-6

**Published:** 2026-10-26

**Authors:** Margaret Slavin, Maryann Greenfield, Anjali Pendala, Valentina A. Andreeva, Ali Ozmeral, Elizabeth Seng

**Affiliations:** 1 Department of Nutrition and Food Science, University of Maryland, College Park, MD USA; 2 School of Medicine, Virginia Commonwealth University, Richmond, VA USA; 3 Nutritional Epidemiology Research Group/EREN, Université Sorbonne Paris Nord, Paris, France; 4 Ferkauf Graduate School of Psychology, Yeshiva University, Bronx, NY USA; 5 Saul R. Korey Department of Neurology, Albert Einstein College of Medicine, Bronx, NY USA; 6 Montefiore Headache Center, Montefiore Medical Center, Bronx, NY USA

**Keywords:** Migraine, Diet, Nutrition, Eating patterns, Gastrointestinal symptoms

## Abstract

**Purpose of Review:**

Diet is frequently cited among the lifestyle interventions important to managing migraine disease, but existing evidence informing dietary recommendations is hindered by limited and heterogeneous dietary study designs and results. Here, we review recent literature on migraine and diet with attention toward study designs needed to better inform dietary recommendations for migraine.

**Recent Findings:**

Numerous recent observational studies agree that dietary intake of individuals with migraine is slightly different than their counterparts without migraine, and the disparity appears to be greater with more severe symptoms. The cross-sectional nature of the data prevents determination of causality. Meanwhile, mechanistic evidence is mounting that the relationship between dietary intake and migraine is bidirectional, yet little has been observed about the *direct* influence of migraine on dietary intake. Increasingly rigorous controlled dietary feeding studies demonstrate the ability of dietary modifications to impact migraine outcomes, and the strongest evidence exists for diets that diverge substantially from typical eating patterns. The implications of long-term dietary intake on migraine prognosis are also not yet known due to lack of prospective data.

**Summary:**

Multidimensional evidence supports the existence of a bidirectional relationship between diet and migraine, but data are lacking on how this interplay actually impacts dietary intake and what it means for dietary recommendations. Future research needs include: expanded study designs to better elucidate how migraine influences diet, clinical trials evaluating interventions that support healthy eating behaviors in the presence of migraine symptoms, and prospective cohorts with longitudinal assessments of migraine and dietary intake, to provide data on the reduction of risk for migraine, its exacerbation and co-morbid diet-related chronic diseases.

## Introduction

The episodic nature and unpredictability of migraine attacks are key defining features of migraine disease. In past decades, a predominance of food-related research in migraine focused on the uncertainty of an individual migraine attack. Research attempted largely to understand whether and how consumption of some key dietary factor precipitates an attack (often studied with the causal implication of a “trigger”), whether by food allergy or some other mechanism, including the more recent hypothesis of acute oxidative stress [1].

In recent years, research on overall eating patterns and habits has increased, attempting to understand a longer-scale relationship by performing an intervention that modifies dietary intake according to a specific pattern and observes impact on migraine disease outcomes (frequency, severity, and disease burden) over time. This shift in the diet-migraine research portfolio has mirrored a paradigm shift in the nutrition field, where evidence-based dietary guidance have shifted away from a focus on individual nutrients or ingredients (i.e., saturated fat or cholesterol) and toward healthy overall dietary patterns (such as the Mediterranean Diet or MyPlate) [2]. While the nutrition field is still concerned with monitoring intake of nutrient adequacy, it is increasingly recognized that relationships between individual nutrients and health outcomes are often non-linear, indicating that balance is key. The change in approach to dietary recommendation supports people in how they eat – not by choosing individual nutrients, but by choosing *foods* in patterns and combinations which inherently supply essential nutrients alongside non-essential bioactive components [3–6]. Some of these components – such as antioxidants, live microbes, and phytochemicals – have also been explored in relation to migraine. This pattern-based approach better supports health while aligning with individual preferences, budgetary constraints, and cultural traditions.

Both acute (i.e., trigger) and long-term (i.e., dietary patterns) implications of diet are of interest as potential points of intervention for migraine. Despite a growing number of publications on the topic of nutrition and migraine in recent years, the field remains challenged to coalesce heterogeneous methodologic approaches to provide meaningful, supportive and actionable evidence-based nutrition recommendations for addressing migraine. This article (1) reviews the state of current, multifaceted data connecting diet and migraine, (2) highlights methodological considerations that limit applicability of current data in making dietary recommendations to patients, and (3) suggests ways to move the science forward for designing patient-centric nutritional interventions for migraine.

## (1) State of the Science: How Confident Can We Be that there *is* a Relationship Between Diet and Migraine?

Evidence from multiple lines of inquiry is converging to give confidence that not only is there a relationship between dietary intake and migraine, but that it is bi-directional [7, 8].

### Diet’s Influence on Migraine

A wide range of randomized clinical trials testing dietary interventions from ketogenic to low lipid, IgG-based elimination diets, and high omega-3 diets have shown benefits for migraine outcomes. These have been reviewed elsewhere and noted considerable variation in study design and quality [9–12]. Increasing rigor of dietary intervention study design, in particular the trials modifying omega-3:omega-6 ratio [13–17], give confidence that *dietary interventions are capable of modulating migraine outcomes*. This confidence is bolstered by corresponding increasing evidence for diet’s ability to improve other/multiple pain conditions [18–20].

Mechanistically, levels of calcitonin gene-related peptide (CGRP)–a neuropeptide involved with migraine pathophysiology–appear to be impacted by individual foods and eating patterns. Evidence is mostly in pre-clinical models, with some supportive evidence that CGRP levels were impacted in small clinical trials of the dietary supplements Vitamin D and curcumin [7, 21]. Emerging data further support a link between dietary behaviors and CGRP signaling; for example, fasting-induced headache has been associated with elevated circulating CGRP levels, while preclinical work suggests CGRP pathways may also influence food-related aversive learning and intake behaviors [22, 23]. Evidence is also building to provide mechanistic explanations for specific food triggers in subsets of patients, including glutamate [24], histamine [25], tyramine, and phenylethylamine, and a potential active component specific to red wine [26]. Also, the role of the gut microbiome–which is influenced significantly by diet [27]–is beginning to come into focus and is increasingly recognized to impact pain, pathogenesis of pain, chronic pain [28, 29] and migraine specifically [30, 31]. Multi-omics data suggest that gut microbiota-metabolome interactions may influence migraine via the gut-brain axis, with downstream effects on CGRP signaling and neuroinflammation [32]. Gut microbes are now being identified through Mendelian randomization for causal associations between gut microbiota and migraine [33, 34]. The gut microbiomes of people with migraine have been associated with inflammation [35] and hypothalamus structure [36] which, as the referenced authors discuss, have been implicated in prior migraine research. Beyond influencing microbiome composition, dietary factors including fiber and polyphenols are well-recognized to influence key aspects of gut health, including inflammation, gut barrier integrity, and gut motility, and thereby direct communication from the gut to the brain via the gut-brain axis [37, 38]. All of these lines of evidence align to support that dietary intake likely influences migraine.

Although dietary triggers (i.e., foods whose consumption precipitates a migraine attack within a short period of time) figure prominently in the literature on migraine, they have been reviewed recently elsewhere [39] and are not the focus here. In short, as new study methodologies arise, evidence against trigger avoidance is building. Some factors, including food cravings, are now suspected to be premonitory symptoms rather than triggers [40]. Evidence further suggests that sensory hypersensitivity, particularly osmophobia, may contribute to the perception of foods as triggers [41]. The use of an “n of 1” design within a proprietary app showed consumption of self-reported food and beverage triggers (caffeine, alcohol, chocolate) was associated with *decreased* migraine risk [42]. Further, blanket recommendations regarding avoidance of potentially triggering foods are inconsistent with the burgeoning literature demonstrating the ineffectiveness and potential unintended negative consequences of emphasizing avoidance as a key migraine management strategy [43].

### Migraine’s Influence on Diet

It has long been understood that the gastrointestinal symptoms and GI co-morbidities of migraine could influence dietary intake [30, 44]. It stands to reason that nausea (a key feature of migraine) impacts appetite and food intake, particularly for those with those with frequent migraine, although the extent or quality of nutritional impact has not been investigated systematically. Furthermore, studies suggest broader alterations in eating behaviors, including reduced food enjoyment, increased hunger responsiveness, and higher rates of maladaptive eating attitudes among individuals with migraine [45, 46].

The neuropeptides highly implicated in migraine pathophysiology, CGRP and pituitary adenylate cyclase activating polypeptide (PACAP) [47] are also likely to impact dietary intake due to their involvement in hunger and satiety signaling [48]. Both CGRP and PACAP have anorexigenic (appetite-suppressing) effects [8, 21, 49]. Meanwhile, the endocannabinoid system is critically involved in both pain processing and eating behavior [50], and its dysregulation has been associated with chronic migraine [51]. Martins-Oliveira et al. present a compelling and complex telling of metabolic disturbances and perturbations in appetite and thirst mechanisms that are likely to influence food intake, including a hypothesis that these mechanisms may conspire to produce a lack of appetite in migraine [8]. If verified, this could potentially explain the higher rates of skipped meals witnessed in observational studies of people with migraine.

Different phases of the migraine attack likely influence food intake in different ways. During the prodromal phase, functional imaging of nitroglycerin-induced attacks have observed hypothalamic activation via fMRI [52], which may drive premonitory symptoms including food cravings [8]. (Notably, limitations in methodology have prompted calls for more rigorous studies [53].) During the headache phase of the attack, the pain itself may influence food intake. Patients report experiencing hunger differently in the presence of physical pain, and that pain (particularly chronic) serves as a stressor which leads to emotional eating and different dietary choices [54–56]. Pain also disrupts hedonic perceptions of highly palatable fat-rich foods [57]. People choose more sweet foods after an induced painful experience than a non-painful experience [58]. This aligns with observations that sweet foods modulate pain sensitivity by increasing the pain threshold [59]. Combined, there is considerable evidence to support that migraine likely influences food intake.

## (2) What are the Methodological Considerations that Currently Limit better Dietary Recommendations for Migraine?

While it is increasingly convincing that the physiology of migraine and pain influences dietary intake *and* that differences in dietary intake impact migraine outcomes, significant uncertainties remain about how to intervene in the diet to improve migraine.

### Data Shows People with Migraine Eat Differently. We Don't Know When This Happens in Relation to Attacks

There is rapidly increasing and diverse observational evidence demonstrating that people with migraine eat differently *overall* than their peers without migraine (Tables 1 and 2), but the nature of the evidence prevents determination of causality, or even direction of the association. These studies use food frequency questionnaires to estimate typical food intake over a set period of time (i.e., a month), and some studies use food diaries or diet recall interviews to calculate average dietary intakes over several days. These are valid and appropriate methods for observing an individual’s typical dietary intake during the prescribed window of time, and they give meaningful insight into the *overall* dietary intake of people with migraine. The sum of the studies in the tables suggest that dietary intake in people with migraine is nutritionally inferior when measured on individual nutrients (Table 1) or food patterns (Table 2), and that the nutrition quality of the diet appears to be worse in the presence of more severe symptoms (Table 3). However, as employed in cross-sectional and case-control study designs, these methods are *not* able to provide insight into the bidirectional nature of the diet-migraine relationship. It is not possible to know with existing data: when people are consuming foods in relation to attacks or phase of attack, whether pain and/or some other migraine symptom(s) drive dietary intake, or to what extent dietary intake is influenced by GI or other symptoms in this population.

**Table 1:** Comparison of Observed Nutrient or Single Component Intake in People with and without Migraine

Reference (Year)	Nutrient	Design	Population	Sample Size	Country	Findings
Pogoda (2016) [96]	Sodium	Cross- sectional	U.S. adults, NHANES* study (age 20+)	8,819	United States	High dietary sodium consumption was associated with lower odds of migraine.
Andreeva (2018) [97]	Macro-nutrients	Cross- sectional	French adults, NutriNet-Santé e-cohort (age 19–64)	31,770	France	Modest gender- specific differences in macronutrient intake were observed between people with and without migraines.
Slavin (2021) [98]	Magnesium	Cross- sectional	U.S. adults age 20–50, NHANES* study	3,626	United States	High dietary magnesium consumption was associated with lower odds of migraine.
Meng (2021) [99]	Magnesium	Cross- sectional	U.S. adults, NHANES* study (age 20+)	10,798	United States	The lowest observed intake of magnesium was associated with increased odds of migraine; the relationship was non-linear.
Meng (2021) [99]	Calcium	Cross- sectional	U.S. adults, NHANES* study (age 20+)	10,798	United States	The lowest observed intake of calcium was associated with increased odds of migraine; the relationship was non-linear.
Meng (2021) [100]	Iron	Cross- sectional	U.S. adults, NHANES* study (age 20+)	7,880	United States	When iron intake is less than 25 mg/day, lower iron intake was associated with increased odds of migraine for women aged 20–50, with no significant association for women over 50 or men.
Li, H (2022) [101]	Riboflavin	Cross- sectional	U.S. adults age 20–50, NHANES* study	3,439	United States	Low dietary and total intake of riboflavin were associated with higher odds of migraine, which when stratified by gender remained significant for females but not males.
Li, D (2022) [102]	Riboflavin	Cross- sectional	U.S. adults, NHANES* study (age 20+)	13,439	United States	No significant relationship was found between dietary riboflavin consumption and migraine.
Li, D (2022) [102]	Thiamin (B1)	Cross- sectional	U.S. adults, NHANES* study (age 20+)	13,439	United States	High dietary intake of thiamin was associated with lower odds of migraine, which when stratified by gender remained significant for females only.
Liu (2022) [103]	Niacin	Cross- sectional	U.S. adults, NHANES* study (age 20+)	10,246	United States	When niacin intake is less than 21 mg/day, lower niacin intake was associated with increased odds of migraine.
Bakirhan (2022) [104]	Polyphenols	Cross- sectional	Adults admitted to an outpatient clinic for episodic migraines (age 19–65)	90	Turkey	Consumption of polyphenols, as flavonones and lignans, were inversely associated with migraine severity.
Huang & He (2023) [105]	Fiber	Cross- sectional	U.S. adults. NHANES* study (age 20+)	12,710	United States	Higher intake of dietary fiber was associated with lower prevalence of migraines, for those with a BMI < 25 or > 30 and those who identified their race as either non-Hispanic white or non-Hispanic black.
Zhang (2023) [106]	Caffeine	Cross- sectional	U.S. adults, NHANES* study (age 20+)	8,993	United States	Higher intake of dietary caffeine was associated with increased odds of migraine, which a subgroup analysis found was significant only in those younger than 60.
Xu (2023) [107]	Potassium	Cross- sectional	U.S. adults, NHANES* study (age 20+)	10,254	United States	When potassium intake is less than 1439 mg/day, lower potassium intake was associated with increased odds of migraine.
Zhao (2024) [108]	Vitamin C	Cross- sectional	U.S. adults, NHANES* study (age 20+)	4,101	United States	Those who consumed the most vitamin C had lower odds of migraine.
Zhao (2024) [109]	Folate	Cross- sectional	U.S. adults, NHANES* study (age 20+)	4,107	United States	Those who consumed the most folate had lower odds of migraine.
Zheng (2024) [110]	Zinc	Cross- sectional	U.S. adults, NHANES* study (age 20+)	9,849	United States	Those who consumed the least zinc had higher odds of migraine.
Cho (2024) [111]	Coffee	Cross- sectional	Korean adults, Circannual Change in Headache and Sleep (CHASE) study (age 20–59)	3,030	Korea	Higher coffee consumption was more common in people with migraine than in people with non-migraine headaches or no headaches.
Yu (2025) [112]	Selenium	Cross- sectional	U.S. adults, NHANES* study (age 20+)	9,849	United States	Selenium intake ≥ 101.9 ug/day was associated with decreased odds of migraine; especially in those age 20–50.
Wu (2025) [113]	Vitamin E	Cross- sectional	U.S. adults, NHANES* study (age 20+)	10,063	United States	Vitamin E intake ≥7.3 mg/day was associated with decreased odds of migraine; the relationship was nonlinear.
Liao (2025) [114]	Caffeine	Cross- sectional	U.S. adults, NHANES* study (age 20+)	5,234	United States	Caffeine intake <97.5 mg/day was associated with decreased odds of migraine; the relationship was nonlinear.
Li (2025) [115]	Magnesium	Cross- sectional	U.S. women, NHANES* study (age 20+)	3,248	United States	Magnesium intake ≥ 325.41 mg/day was associated with decreased odds of migraine in pre-menopausal women.
Tang (2025) [116]	Alcohol	Cross- sectional	U.S. adults, NHANES* study (age 20+)	13,083	United States	Those who consumed the most alcohol had lower odds of migraine.
Gao (2025) [117]	Milk	Cross- sectional	U.S. adults, NHANES* study (age 20+)	8,850	United States	Those who consume skim milk had lower odds of migraine than those who consume whole milk
Lucerón-Lucas-Torres (2025) [118]	Wine	Meta- analysis of cross- sectional	People in United Kingdom, Norway, United States, and Spain (complete age range not reported)	60,144	Spain	No significant association between wine consumption and odds of migraine.

*NHANES = National Health and Nutrition Examination Survey

**Table 2:** Comparison of Observed Dietary Patterns in People with and without Migraine

Reference (Year)	Food / Pattern	Design	Population	Sample Size	Country	Findings
Ariyanfar (2019) [119]	Fruit and Vegetables	Case- control	Control: children Cases: children with migraine (age 7–14)	190 controls, 100 cases	Iran	High fruit and vegetable intake was associated with lower odds of migraine in pediatrics and adolescents.
Mansouri (2020) [120]	Dairy	Cross- sectional	University students (age 18+)	83,622	Iran	The odds of primary headaches were lower for those with high dairy intake.
Mousavi (2021) [121]	Dietary Acid Load	Case- control	Control: general population; Cases: People with migraine recruited from headache clinics (age 18–60)	582 controls, 514 cases	Iran	High dietary acid load was associated with greatly increased odds of migraine. Dietary acid loads were not significantly different between people with chronic and episodic migraine.
Mansouri (2021) [122]	Fruit and Vegetables	Cross- sectional	University students (age 18+)	83,214	Iran	The odds of primary headaches were lower for those with high fruit and vegetable intake. The association was stronger for fruits among females and for vegetables among males.
Martins (2022) [123]	Dietary Inflammatory Index (DII)	Case- control	Control: healthy adults Cases: adults with episodic migraine (age 18–65)	62 controls, 90 cases	Brazil	Participants with migraine were found to have lower HEI scores and higher DII scores than controls. These scores were not associated with frequency or severity of migraine.
Ariyanfar (2022) [124]	Dairy	Case- control	Control: children Cases: children with migraine (age 7–14)	190 controls, 100 cases	Iran	High intake of low-fat dairy was associated with lower odds of migraine in pediatrics and adolescents.
Liu (2023) [125]	Dietary Inflammatory Index (DII)	Cross- sectional	U.S. adults, NHANES* Study (age 20+)	10,359 (2083 with severe headache or migraine, 8276 without)	United States	Higher dietary inflammatory index scores were associated with increased odds of migraine or severe headache.
Martami (2023) [126]	“Western” and “prudent” patterns	Case- control	Control: adults from outpatient clinic Cases: adults with migraine (age 18–60)	250 controls, 250 cases	Iran	High adherence to the prudent dietary pattern was associated with lower odds of migraine. High adherence to the Western dietary pattern was associated with higher odds of migraine.
Fotros (2024) [127]	Healthy Eating Index − 2015	Case- control	Control: adults from general population Cases: adults with migraine (age 18+)	512 controls, 476 cases	Iran	No significant trend between HEI and odds of migraine, although significant differences between people with migraine and people without migraine were observed for multiple component food groups.
Selen (2025) [128]	Mediterranean, Mediterranean- DASH Diet Intervention for Neurodegenerative Delay (MIND), DII	Case- control	Control: adults in same geographic area (age 18–45) Cases: adults with newly diagnosed migraine (age 18–45)	60 controls, 84 cases	Türkiye	Higher adherence to Mediterranean and MIND diets, and higher DII score associated with lower odds of migraine.
Zuo (2025) [129]	Composite Dietary Antioxidant Index (CDAI)	Cross- sectional	U.S. adults age 20–50, NHANES* study	9190	China	The association between CDAI and odds of migraine followed a U-shaped pattern, with an inflection point of 0.2 CDAI found to have the lowest odds of migraine.

*NHANES = National Health and Nutrition Examination Survey; CDAI = Composite Dietary Antioxidant Index (CDAI); DASH = Dietary Approaches to Stop Hypertension; DII = Dietary Inflammatory Index; HEI = Healthy Eating Index; MIND = Mediterranean-DASH Intervention for Neurodegenerative Delay

**Table 3:** Observed Dietary Patterns in Relation to Migraine Severity in People with Migraine

Reference (Year)	Food / Pattern	Design	Population	Sample Size	Country	Findings
Askarpour (2020) [130]	MIND	Cross- sectional	Female neurology clinic patients diagnosed migraine (age 18–50)	266	Iran	Higher adherence to MIND dietary patterns was associated with lower intensity, duration, and frequency of migraine, but not associated with migraine related disability.
Togha (2021) [131]	Data Driven patterns	Cross- sectional	Female neurology clinic patients with migraine (age 18–50)	256	Iran	The PCA derived “healthiness” of dietary patterns was associated with lower migraine severity, but not migraine related disability.
Bakirhan (2021) [132]	DASH & Mediterranean Diet	Cross- sectional	Adults (age 18–64) with neurologist-diagnosed episodic migraine	80	Turkey	The following patterns were associated with lower severity of migraine attacks: high diet quality; consumption of vegetables, fruits, legumes, and seed oils. DASH and Mediterranean dietary patterns were associated with lower severity and frequency of migraine attacks.
Arab (2023) [133]	Mediterranean	Cross- sectional	Adults (age 20–50) with diagnosed migraine	262	Iran	Higher adherence to the Mediterranean diet was associated with lower frequency, duration, and disability of migraines.
Tirani (2023) [134]	Dietary Diversity	Cross- sectional	Neurology clinic patients diagnosed with migraine (age 20–50)	262	Iran	Higher levels of diet diversity was associated with lower frequency of migraine, along with lower serum levels of nitric oxide.
Balali (2024) [135]	Healthy Eating Index 2015 + Alternative HEI	Cross- sectional	Neurology clinic patients with migraine (age 18–50)	262	Iran	High scores for HEI and alternative-HEI were both associated with lower migraine attack frequency. High alternative-HEI was also associated with lower migraine disability.
Hajjarzadeh (2024) [136]	Total antioxidant capacity	Cross- sectional	Females with diagnosed migraine (age 25–55)	289	Iran	Lower migraine attack frequency was associated with diets with higher antioxidant capacity.
Hajjarzadeh (2024) [137]	DASH (Dietary Approaches to Stop Hypertension)	Cross- sectional	Females with diagnosed migraine (age 25–55)	285	Iran	Higher DASH score was associated with lower frequency and severity of migraine attacks.
Bakhshimoghaddam (2024) [138]	Dietary Inflammation Score	Cross- sectional	Female neurology clinic patients with migraine (age 25–55)	285	Iran	High dietary inflammation score was associated with higher odds of chronic migraine (15 or more migraine attack days per month)
Jebraeili (2024) [139]	Carbohydrate Quality Index	Cross- sectional	Female neurology clinic patients with migraine (age 18–45)	266	Iran	High carbohydrate quality index was associated with lower migraine pain severity and duration of headache.
Arabshahi (2024) [140]	Glycemic Index / Glycemic Load	Cross- sectional	Female neurology clinic patients with migraine (age 18–50)	266	Iran	High glycemic index and glycemic load were associated with higher migraine severity and disability.
Mittleman (2024) [141]	Habitual caffeinated beverages	Prospective cohort	Adults with episodic migraine (age 18+)	97	United States	Baseline caffeine intake was not associated with headache frequency, severity, or duration over 6 weeks of observation.
Hajishizari (2024) [142]	Dietary Antioxidant Quality Score (DAQS)	Cross- sectional	Female neurology clinic patients with migraine (age 18–50)	265	Iran	High DAQS was associated with lower headache frequency. Adherence to the vitamin C component of DAQS was associated with lower migraine pain severity.
Esteves-Mesquita (2025) [143]	Food groups, specific foods	Cross- sectional	Adults with migraine (age 18–64)	260	Spain	No differences observed for meat, dairy, fruits, vegetables, or grains. High caffeine intake was associated with lower migraine attack frequency. High alcohol intake was associated with lower attack frequency and migraine related disability. High cured meat and cured cheese were associated with lower migraine related disability.

DASH = Dietary Approaches to Stop Hypertension; DAQS = Dietary Antioxidant Quality Score; HEI = Healthy Eating Index; MIND = Mediterranean-DASH Intervention for Neurodegenerative Delay; PCA = Principal component analysis.

Accurate assessment of dietary intake is challenging due to participant and researcher time burden, dependence on self-report and memory, the potential for the act of recording intake to influence food choices, the chemical complexity of food, and variation in nutrient content and composition across the food supply. Tools are available to minimize these concerns, but each project requires a nuanced selection and application of the best dietary assessment method [60]. Some apps and headache diaries are used to track suspected individual food triggers, but there is currently no reliable, consistent method for measuring *detailed* dietary intake in temporal proximity to migraine. We have demonstrated on a pilot level that pairing intermittent 24-hour recalls with daily headache diaries can detect meaningful within-person, day-level shifts in dietary intake across migraine phases [61]. Without understanding the influence of the disease symptoms on dietary intake, it is difficult to design nutritional interventions that would promote nutritional health and be attainable by people experiencing relevant symptoms such as pain, nausea, or fatigue.

### Diet Interventions Work for Migraine in Very Controlled Study Designs. We Don’t have Sufficient Data to Show this Translates to Real-World Settings

To establish biological plausibility of a dietary hypothesis (i.e., *Does a _____ diet decrease migraine frequency? )*, the tightest control of dietary intake is used within a controlled feeding trial. Most or all of participants’ food is provided to them for the trial duration, sometimes in combination with frequent dietary counseling. This provides the greatest chance of observing the hypothesized biological and clinical outcomes, without relying on participants’ ability to obtain and prepare their own food in the appropriate patterns. While maximizing scientific validity, this strict design inherently suffers from concerns about ecological validity, because it is far from how people eat in the real world and not sustainable after the trial ends.

Thus far, most dietary trials conducted for migraine are considered to be low quality by common bias-rating schemas [9]. The trials with the strongest design—and therefore strongest evidence of biological plausibility for dietary interventions improving migraine—are diets a high omega-3, low omega-6 intervention or solely low omega-3 interventions [13–17]. The high omega-3 low omega-6 intervention had the higher effect size, but is far from the typical dietary fat intake profile in western societies, making it difficult to implement in daily life and often not sustainable. The ketogenic diet was the subject of a recent meta-analysis combining data from 10 studies, although most are open-label or prospective observational [62]. The one double-blind controlled trial was small but did show benefit of the very low calorie ketogenic diet, as compared to the very low calorie non-ketogenic diet [63].

It is challenging to change one’s eating pattern, often requiring people to modify central aspects of daily life (i.e., shopping, family meals, eating out, social engagements, cultural traditions, and so forth). In contrast to a fully-controlled feeding study, a dietary counseling trial is a more real-world investigation that tests delivery of dietary counseling as in clinical practice and in the context of participants’ usual lives [64]. Participants are taught and coached to make decisions on procuring, preparing, and eating food types and quantities aligned with some desirable dietary pattern. This trial design therefore tests the effect of an *achievable* dietary change, with counseling support. (i.e., *Does dietary counseling for the _____ diet decrease migraine frequency?*)

Few studies have used adequately designed dietary counseling interventions to evaluate improvements in migraine symptoms. Artificial intelligence (AI)-enabled platforms may help deliver scalable, personalized dietary counseling. One study provided 15 min of nutrition education and written materials as the intervention before a 12-week observation period [65]. The ketogenic trials employing a nutrition counseling/education approach had a high dropout rate, surpassing 30%, although several ketogenic studies did not clearly differentiate how the diet intervention was delivered (provision of food vs. nutrition counseling) or if dietary counseling occurred more than once [62].

Low-glycemic diet studies are possibly the closest to this approach, and are showing promise for migraine prevention in a combination of prospective observational studies and one trial using a digital therapeutic pairing continuous blood glucose monitoring with a proprietary app to make personalized nutrition recommendations for blood glucose control. Two prospective studies using the digital therapeutic for 16 weeks observed a decrease in migraine days [66]. Recently, a large (*n* = 842) open-label trial of the same app showed greater decreases in migraine days than the placebo app [67]. An open-label, but lower quality low-glycemic counseling trial *without* continuous glucose monitoring also showed decreases in migraine frequency [68].

Additional controlled feeding trial *and* dietary counseling trial evidence is needed for achievable diets (i.e., low glycemic, Mediterranean diet, high fruits/vegetables) to test whether the diets work for migraine *and* in the real world. Multi-arm investigations where dietary counseling is one of several components of a comprehensive lifestyle intervention trial, possibly alongside stress management, sleep hygiene, and physical activity [62]. Pairing investigations of patient experiences with dietary intervention trials (as in this trial exploring patient experiences with the ketogenic diet for pain) [69] would provide clinicians with critical information needed for identifying barriers and supports for patients undertaking these diet and lifestyle changes.

### The Timing of Food Intake is Related to Migraine Attacks. We Don't Know if or How to Intervene

Timing and regularity of food intake is among the eating patterns of interest to migraine, and it is well-accepted that extended fasts are capable of triggering attacks. Over 40% of participants affirm that they perceive missing a meal to provoke migraine attacks by questionnaire [70, 71]. Self-reported regular breakfast eaters were less likely to have a primary headache, as compared to those who regularly skipped breakfast in a large population study [72]. Higher incidence of migraine is observed following the natural experimentation offered by religious fast for Ramadan [73, 74], similar to what has been observed for Yom Kippur, with a recent study further indicating increased migraine frequency and headache burden during Ramadan fasting [75]

Because of these observations and general guidance aimed at maintaining consistent routines for migraine prevention, it is commonly recommended for people with migraine to consume consistent meals, but there is limited direct evidence to support this recommendation. Fasting and meal skipping are commonly reported triggers, although sustained fasting states that induce ketosis may reduce both the frequency and disability associated with migraine [76]. Observational data suggest migraine burden peaks early during fasting periods, supporting the concept that disruptions in expected meal timing may act as “surprisal” triggers. The anti-orexigenic properties of CGRP and PACAP may explain meal skipping as the result of the early stages of an attack – when these peptides are elevated, individuals may simply lose appetite and not eat [8]. Meal skipping and migraine should also be evaluated with an eye toward food insecurity, since people experiencing food insecurity have limited or uncertain access to food. In 2023, 13.5% of U.S. households were food insecure [77]. The prevalence of migraine was higher in young adults with food insecurity [78].

Analyses of prospective data from a migraine tracking app found that days with a missed meal were associated with an increased risk of migraine attack onset for a small proportion (8.2%) of participants [42]. Further research is needed to separate if meal skipping may be a symptom, instead of or in addition to a trigger, and for whom. Studies precisely controlling dietary timing would be difficult to conduct with ecological validity and unlikely to provide meaningful insight. Novel designs intended to minimize meal skipping, enhance snacking, or reduce duration of overnight fasts may be options to approach this question.

### Diet is a Major Influence on Long-Term Health. We Have Very Little Data to Connect Typical Eating Patterns with Long-Term Migraine Prognosis

There is essentially no information on whether long-term diet patterns may serve to prevent migraine or prevent its exacerbation or chronification. To understand the long-term influence of eating patterns, prospective cohort studies observing both migraine and food intake are needed. Despite past criticisms for imprecision and potential for bias of epidemiological designs as compared to RCTs, recent meta-epidemiological studies comparing the conclusions of pooled RCTs against pooled cohort studies have found only small differences in results of diet-disease outcomes [79, 80], suggesting that cohort studies are not only a valid but a necessary approach for nutrition research, given that they often fill a gap that cannot be addressed through RCTs.

Large, diverse general population prospective cohort studies would be useful in studying nutritional influence on migraine disease incidence in the general population, in modeling the effect of multiple dietary exposures or controlling for a wide range of confounders (gender, socioeconomic status, ethnicity, BMI). Prospective cohorts can also explore questions that cannot ethically be considered by RCTs (i.e., the influence of nutritional deficiencies or unhealthy dietary patterns on prognosis), in performing analyses on different subgroups (i.e., different types of migraine, by symptom clusters, by gender or life stage), and in explaining relationships of migraine and diet and diet-related co-morbidities (i.e., diabetes, cardiovascular disease, obesity) that cannot be understood on the shorter timescale of intervention trials. Prospective cohorts can also serve as a platform for nested case-cohort and case-control studies [81], and support the generation of targeted hypotheses worthy of the limited resources available for future dietary RCTs. RCTs, which are more feasible for dietary components such as supplements, have demonstrated benefit for select dietary factors in migraine. Results from multiple cohorts can be pooled to add statistical power, observe a wider range of dietary exposures, and include a more diverse population [82]. Ultimately, prospective cohort studies in nutrition are valuable in diet-disease investigations, providing some evidence for causality. Inclusion of migraine outcomes in future health and diet cohorts is critical to understanding if diet plays any role in onset of disease or progression.

For those with existing disease, narrower disease-specific cohorts which measure diet prospectively could provide data on diet’s influence on disease progression and mitigation, and enable deployment of specifically developed tools/questionnaires or collection of biospecimen for metabolite comparisons. Yet, dietary data has not been commonly included in migraine cohorts, likely because of the high time demand for completion of dietary assessments.

## 3) Existing Nutritional Interventions are Novel to Migraine and Hold Promise to Reduce Symptoms and Improve Quality of Life

### How Can We Learn From Existing Nutritional Therapies?

Nausea and vomiting are not specific to migraine. Indeed, over 15 clinical trials have contributed to existing guidelines for dietary management of chronic nausea and vomiting or gastroparesis. These interventions have yet to be evaluated to address nausea and vomiting, or gastroparesis, in a migraine population.

Nutritional treatment of chronic nausea and vomiting could include multiple small-volume meals per day with an emphasis on liquid nutritional sources and small particles for solid foods, as well as restricted fats and dietary fiber [83]. Medical nutrition therapy has also not been evaluated in migraine, which consists of seeing a Registered Dietitian Nutritionists who would tailor recommendations for specific symptoms of gastroparesis, nausea and vomiting, while also paying attention to maintaining fluid balance, nutritional status, medications, and preventing development of learned food aversions that occur with vomiting [84, 85]. These interventions have demonstrated efficacy in other conditions associated with nausea and vomiting, most notably chemotherapy-induced nausea and vomiting [86]. Similar support for individuals with migraine may support both symptom management and reduce nutrition risk.

Gastroparesis is a component of nausea/vomiting in migraine and has a substantial impact on quality of life. The clinical guidelines on gastroparesis from the American College of Gastroenterology recommend dietary modifications including small particle size, low fat, and easily digestible foods to limit symptoms and promote gastric emptying [87]. To date, no studies have evaluated these types of strategies to reduce gastroparesis in migraine. Clinical trials are warranted to investigate whether similar interventions for managing GI-related migraine symptoms could reduce GI-related migraine symptoms, other migraine symptoms, and improve quality of life.

## 4) Study Design Considerations

Given the paucity of current evidence, diverse study designs, including “surprisal”-based trigger metric [88], standardized outcome frameworks (e.g., IHS migraine control tiers), and AI-enabled approaches to modeling complex dietary exposures, will be essential to inform optimal dietary recommendations for migraine. Any dietary intervention must be attainable and affordable within the existing food system, and match the preferences, norms and values of the individual. Designing dietary interventions for migraine is uniquely complex due to the bidirectional interaction between diet and migraine symptoms, with recent studies increasingly evaluating mental health outcomes alongside headache measures. For an eating pattern to be sustainable for individuals with migraine, it also needs to be responsive and adaptable to their dynamic migraine symptoms. People with migraine may also present with relevant comorbidities (such as obesity, cardiovascular disease, IBS), which may need to be considered an exclusion criteria for clinical trials, and certainly should be considered in the development of a clinical dietary intervention for an individual patient.

Adherence to healthy diets is challenging in the current food environment. Providing all, or a portion, of the food that one is expected to eat is one way to improve adherence with a particular diet and reduce decision fatigue. For example, the Food as Medicine approach, provides individuals with a substantial portion of their food, similar to a medical prescription [89]. Similar approaches have proved cost-effective for treatment of diabetes, reducing complications and prescriptions [90]. Future clinical trials for migraine could consider using this type of approach; however, a challenge with migraine is that nutrition needs may change based on unpredictable symptoms that can’t be anticipated prior to food delivery. Food as Medicine may be another area where AI may enhance personalization, allowing dynamic dietary prescriptions based on symptoms patterns and real-time data.

Wearables offer interesting new possibilities. Continuous blood glucose monitoring (CGM) alongside dietary intake monitoring may be able to resolve some uncertainty relating to the influence of food timing or mealtime regularity on attacks and help personalize dietary approaches to match individualized metabolic response to variations in food intake, particularly macronutrients. The development of support for CGM to help personalize dietary recommendations for migraine has followed increasingly vigorous study designs, from a retrospective proof-of-concept [91], to a prospective cohort [66], and proceeding to a protocol for a randomized controlled trial for a medical device [67]. Recent work further supports this approach, demonstrating that individuals with chronic migraine exhibit greater glycemic variability and postprandial dysregulation compared to controls, suggesting CGM may help identify metabolically distinct migraine phenotypes and guide personalized dietary strategies [92]. Digital alternatives to CGM, such patch or wristband sensors, may provide similar insights [93]. Wearables are a potentially well suited application for AI, as has been suggested from both nutrition [94] and headache [95] perspectives. Future diet clinical trials in migraine should evaluate the potential utility of CGM as a decision tool.

## Conclusions

Nutritional research in migraine disease is coalescing to show that there is a relationship: diet is able to impact migraine and vice versa. By influencing food choices, migraine has the potential to negatively influence risk of diet-related chronic diseases, like diabetes, obesity, and cardiovascular disease. And people with migraine may have a difficult time following dietary recommendations because of their symptoms.

Still, methods and designs have not yet begun to *understand* the bidirectional relationship, and what it may mean for dietary recommendations for patients who would carry the burden of implementing dietary changes. Considerable evidence is needed before broad recommendations can be made on the use of dietary interventions in the management of migraine. In this regard, we need more RCTs of controlled feeding trials that test the efficacy of more readily attainable dietary patterns *and* the RCTs of behavioral interventions aimed at achieving a desirable eating pattern in the real world. Further, long-term, longitudinal prospective cohort studies connecting diet and migraine outcomes have the potential to change the field of migraine nutrition research by contributing data that would inform on diet’s ability to influence migraine progression. Ultimately, to untangle the complex interplay between migraine and food, the field must adopt different methodologies and study designs, and in some cases develop new ones.

## Key References

- Martins-Oliveira M, Tavares I, Goadsby PJ. Was it something I ate? Understanding the bidirectional interaction of migraine and appetite neural circuits. Brain Res. 2021;1770:147629. https://doi.org/10.1016/j.brainres.2021.147629
  - ○ This review paper presents a complex and compelling description of metabolic connections between migraine pathophysiology, energy metabolism, and appetite regulation, particularly as mediated by signaling peptides CGRP, PACAP, orexins, insulin, glucagon, neuropeptide Y, and leptin. Multiple opportunities for enhancing future research and identifying new therapeutic targets are described. A new hypothesis emerges: meal-skipping commonly observed in migraine may represent a premonitory symptom of migraine due to loss of appetite, rather than a trigger of an attack.
- Evers S, Grube HCB, Gendolla A, Gaul C, Ewald K, Witt O, et al. Efficacy of digital therapeutic sinCephalea for personalised nutrition versus control for migraine prevention: A 12-week open-label randomised clinical trial. Cephalalgia. SAGE Publications Ltd STM; 2026;46:03331024261459744. https://doi.org/10.1177/03331024261459744
  - ○ This study presents results from an open-label clinical trial of wearable continuous glucose monitor paired with a digital therapeutic app which personalizes low-glycemic nutrition recommendations for patients with migraine. The digital intervention resulted in fewer migraine days than the design-matched control. This represents a new research direction for scalable personalized nutrition recommendations for migraine.
- Greenfield MR, Frankenfeld C, Pendala A, Ozmeral A, Pendala V, Baustian N, et al. Variations of Dietary Intake Across Migraine Phases in Adults with Episodic Migraine: A Prospective Observational Pilot Study. Curr Dev Nutr. 2026;10:107668. https://doi.org/10.1016/j.cdnut.2026.107668
  - ○ This article presents a novel study design for observing diet in migraine patients, using a case-crossover design with daily headache diaries and intermittent dietary interviews to maintain high quality of dietary data collection during persistent monitoring. Pilot results showed differences in protein intake across migraine phases and lower nutritional quality of foods eaten on headache days with high pain compared to low pain days. This represents a new direction for understanding food intake in relation to migraine attacks.

## Data Availability

No datasets were generated or analysed during the current study.
